# Supplementary material for: Structural basis for the type I-F Cas8-HNH system
Source: EMBO J. 2024 Sep 9;43(20):4656–67. doi: 10.1038/s44318-024-00229-8 (PMC11480323; doi:10.1038/s44318-024-00229-8)
Supplement: Supplementary file 1 — Appendix [file 44318_2024_229_MOESM1_ESM.pdf]

Appendix for

Structural basis for the type I-F Cas8-HNH system

Xuzichao Li[1, 2], Yanan Liu[3], Jie Han[1,4], Lingling Zhang[1], Zhikun Liu[1], Lin Wang[1], Shuqin Zhang[1], Qian Zhang[1], Pengyu Fu[5], Hang Yin[5], Hongtao Zhu[3]*, Heng Zhang[1, 2]*

1. Tianjin Institute of Immunology, State Key Laboratory of Experimental Hematology, International Joint Laboratory of Ocular Diseases (Ministry of Education), Key Laboratory of Immune Microenvironment and Disease (Ministry of Education), The Province and Ministry Co-sponsored Collaborative Innovation Center for Medical Epigenetics, School of Basic Medical Sciences, Tianjin Medical University, Tianjin 300070, China.
2. Department of Biochemistry and Molecular Biology, Tianjin Key Laboratory of Cellular Homeostasis and Disease, School of Basic Medical Sciences, Tianjin Medical University, Tianjin, China.
3. Beijing National Laboratory for Condensed Matter Physics, Institute of Physics, Chinese Academy of Sciences, Beijing, China.
4. Department of Anatomy, School of Basic Medical Sciences, Tianjin Medical University, Tianjin, China.
5. Department of Pharmacology, School of Basic Medical Sciences, Tianjin Medical University, Tianjin 300070, China.
*Correspondence: hongtao.zhu@iphy.ac.cn (H.Z.), zhangheng134@gmail.com (H.Z.)

Table of Contents

Appendix Figure S1 .... **2**
Appendix Figure S2 .... **3**
Appendix Figure S3 .... **4**
Appendix Figure S4 .... **5**
Appendix Figure S5 .... **6**
Appendix Figure S6 .... **7-8**
Appendix Figure S7 .... **9**
Appendix Figure S8 .... **10**
Appendix Figure S9 .... **11**
Appendix Figure S10 .... **12**
Appendix Figure S11 .... **13**
Appendix Figure S12 .... **14**
Appendix Figure S13 .... **15**
Appendix Figure S14 .... **16**
Appendix Table S1 .... **17**

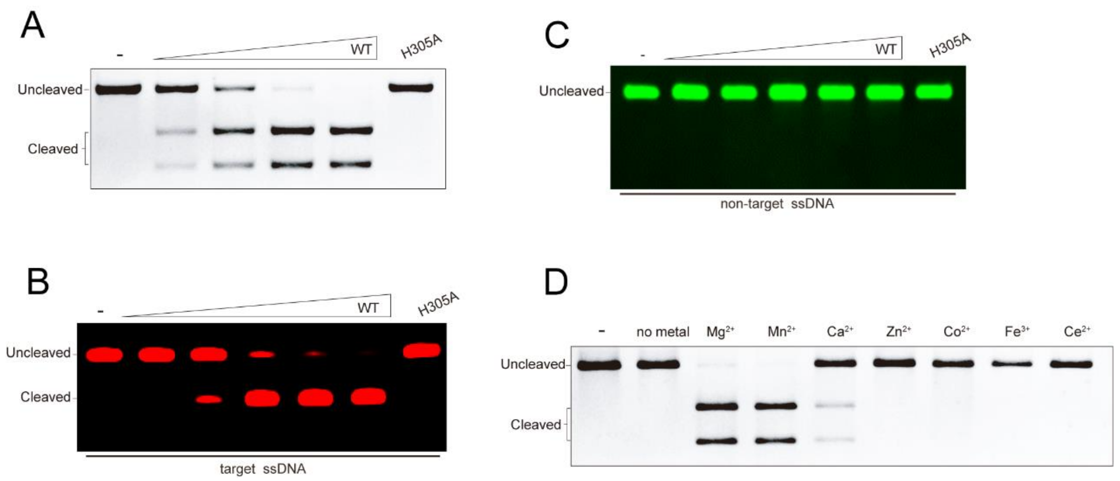

Appendix Figure S1. In vitro cleavage assay of type I-F Cas8-HNH system.

(A) In vitro DNA cleavage assay of type I-F Cas8-HNH system with increasing concentration of the RNP complex. **(B)** In vitro ssDNA cleavage assay of Cas8-HNH system complex with increasing concentration of the RNP complex. Single-stranded targeting DNA substrate was labeled with 3'-Cy5 fluorophore. **(C)** In vitro ssDNA cleavage assay of WT and mutated Cas8-HNH system utilizing 5'-Cy3 labeled ssDNA. **(D)** In vitro DNA cleavage assay of Cas8-HNH system in the presence of different metal ions. For **A-D**, all the experiments were replicated at least three times, and the gels represent three repeat experiments.

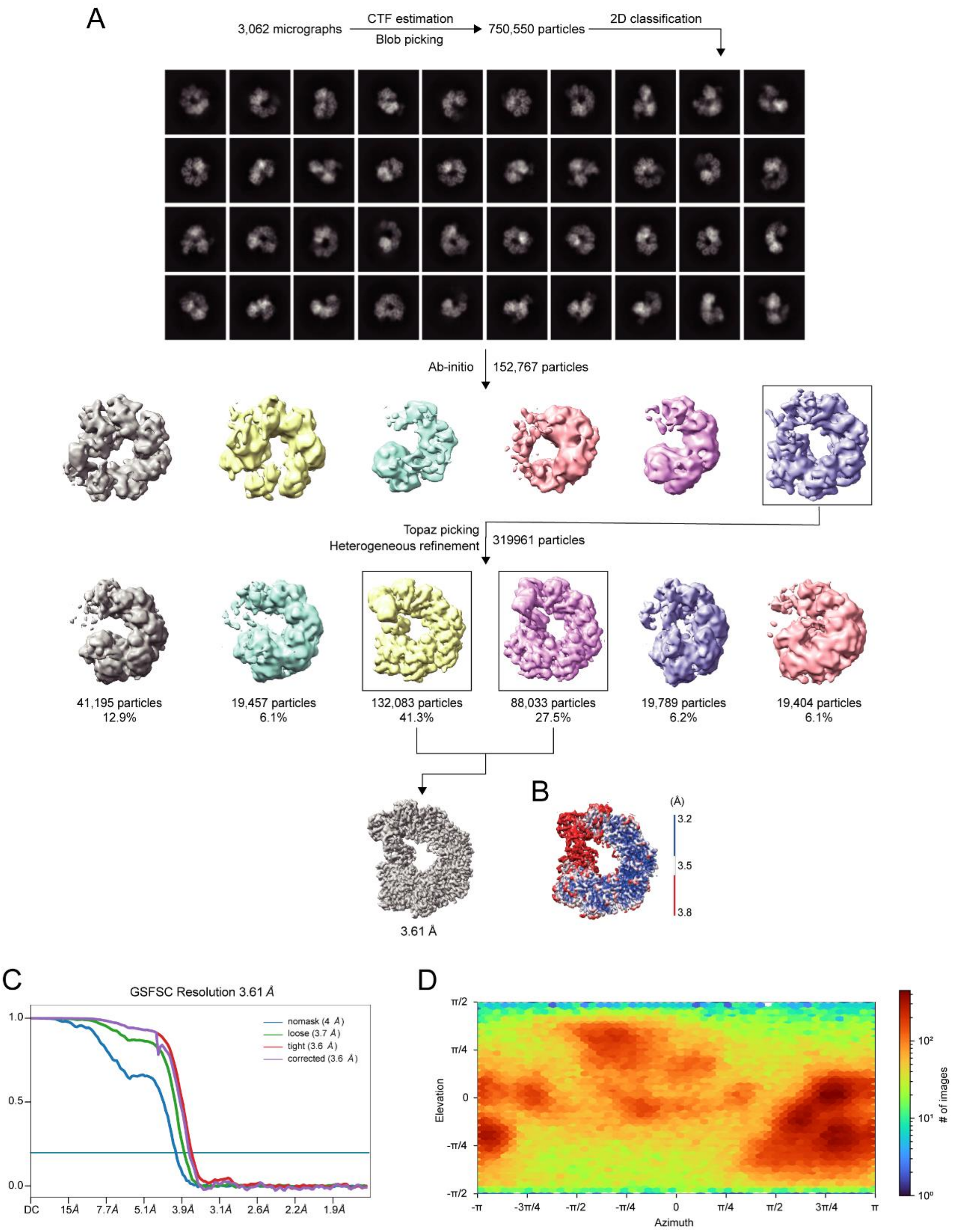

Appendix Figure S2. Cryo-EM data processing workflow of Cas8-HNH RNP complex.
(A) Data processing workflow of Cas8-HNH RNP complex. **(B)** Local resolution analysis. The map of RNP complex colored by local resolution is shown. **(C)** GSFSC plot of the reconstruction. The final resolution was estimated based on the FSC=0.143 cutoff. **(D)** Orientation distributions of the refined particles.

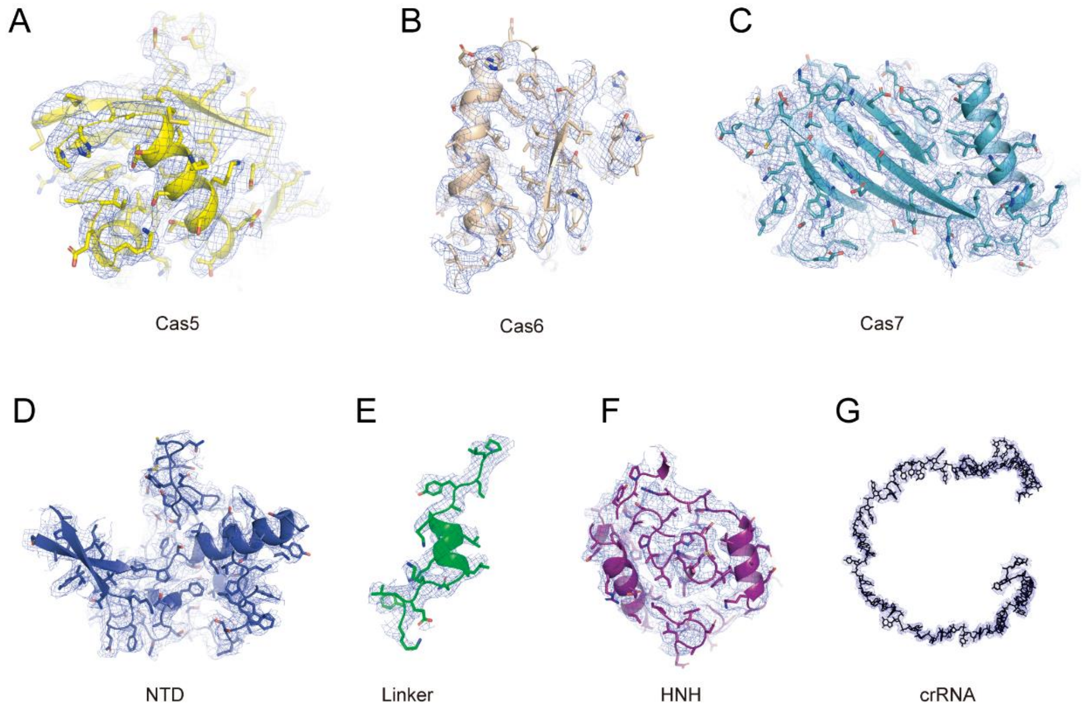

Appendix Figure S3. Representative atomic models of protein subunits and crRNA fitting into the corresponding cryo-EM density map.

(A-C) Cryo-EM density maps and refined models for Cas5, Cas6 and Cas7 subunits. The models of Cas5, Cas6 and Cas7 proteins are shown in yellow, wheat and teal, respectively. **(D-F)** Cryo-EM density maps and models for N-terminal domain (NTD, blue), HNH domain (purple), and the Linker (green) of Cas8-HNH protein. **(G)** Cryo-EM density maps and models for crRNA (black).

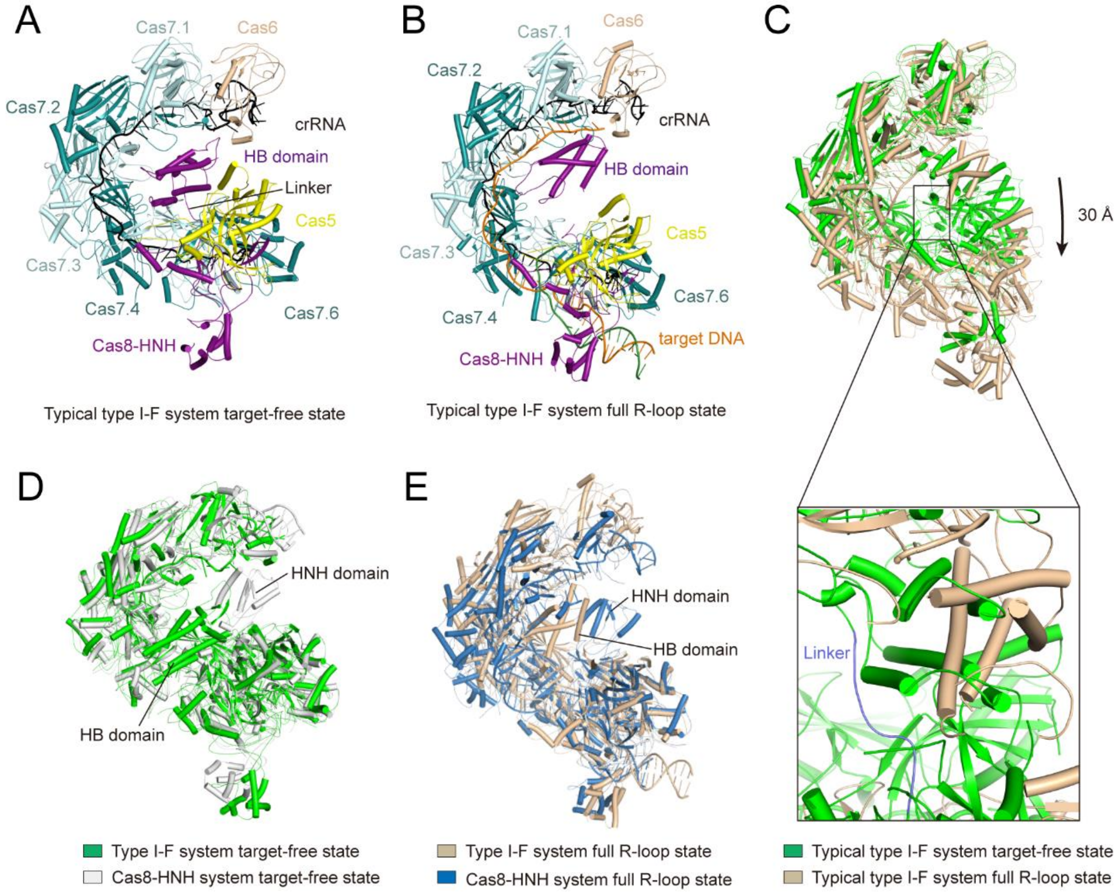

Appendix Figure S4. Structural comparison of canonical type I-F system and Cas8-HNH system.

(A-B) Atomic models of type I-F system target-free (PDB: 6B45) and full R-loop (PDB:6NE0) states. Cas6, Cas5, Cas7, and Cas8 subunits are shown in the same color scheme as in Fig. 2A. **(C)** Structure alignments of canonical type I-F system in target-free (green) state and that in full R-loop (wheat) state. The Linker is shown in slate (lower panel). **(D)** Superposition of canonical type I-F system (green) and Cas8-HNH system (gray) in target-free states. **(E)** Superposition of typical type I-F system (wheat) and Cas8-HNH system (sky blue) in full R-loop states.

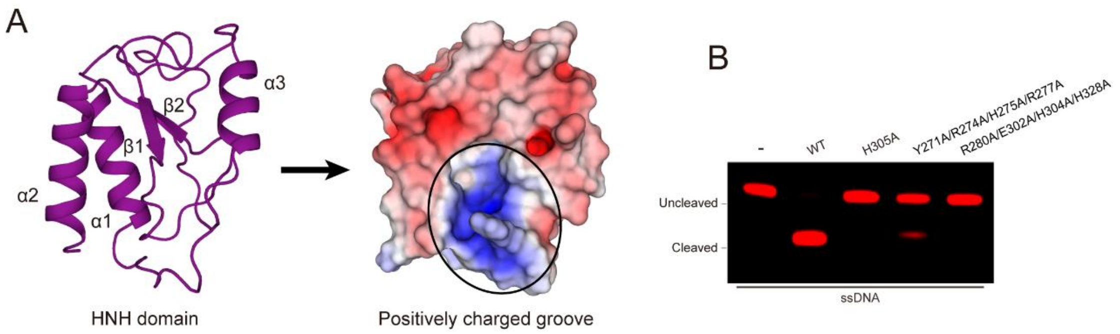

Appendix Figure S5. The potential active-site of the HNH nuclease domain.

(A) Electrostatic surface potential of HNH nuclease domain. The positively charged binding pocket marked by a black circle might be responsible for target DNA binding. **(B)** In vitro ssDNA cleavage assay of WT and mutant Cas8-HNH Cascade utilizing 3'-Cy5 labeled ssDNA. Charged and aromatic residues on the positively charged binding groove of HNH domain are mutated. The gel represents for three independent and repeat experiments.

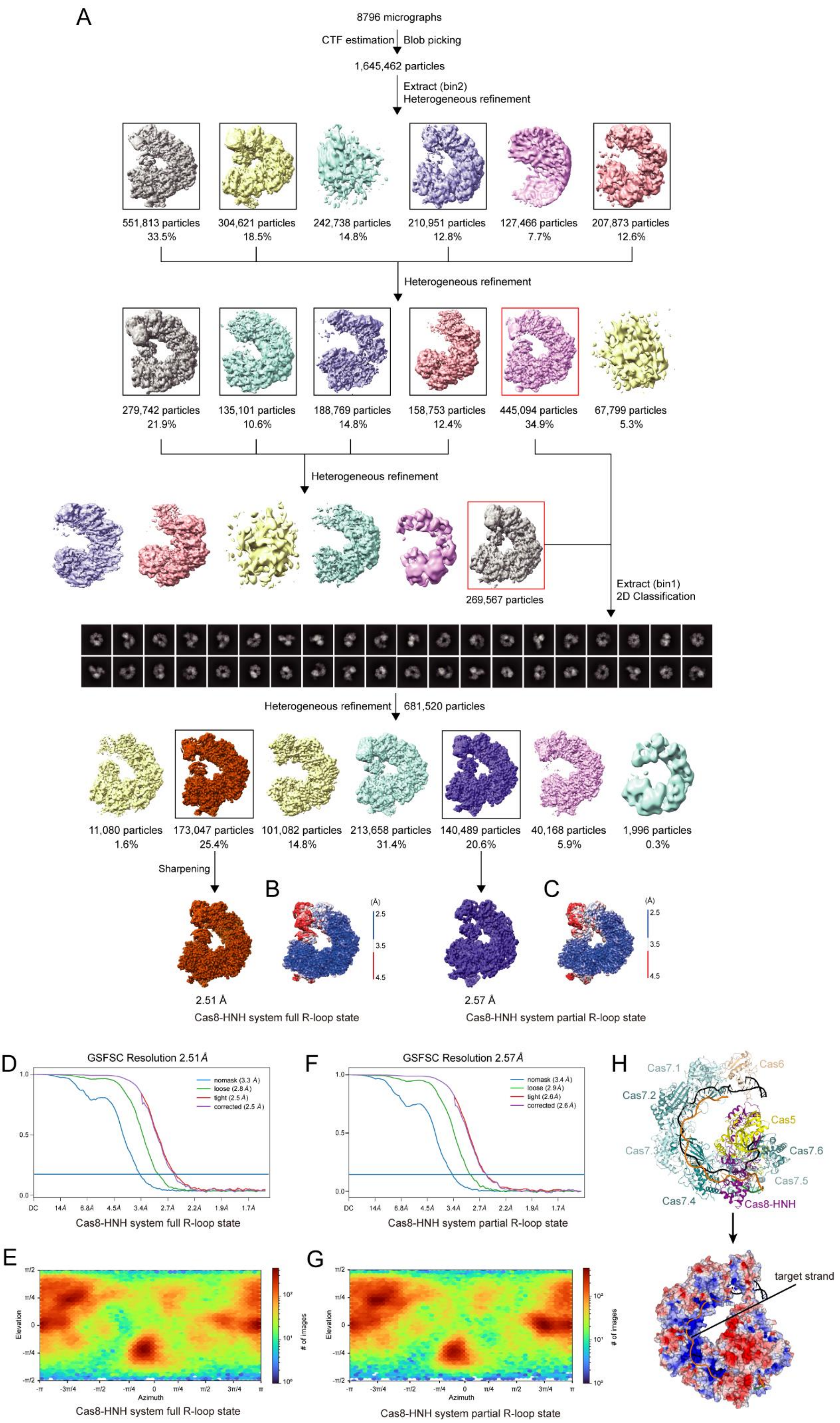
A
8796 micrographs
CTF estimation
Blob picking
1,645,462 particles
Extract (bin2)
Heterogeneous refinement
551,813 particles
33.5%
304,621 particles
18.5%
242,738 particles
14.8%
210,951 particles
12.8%
127,466 particles
7.7%
207,873 particles
12.6%
Heterogeneous refinement
279,742 particles
21.9%
135,101 particles
10.6%
188,769 particles
14.8%
158,753 particles
12.4%
445,094 particles
34.9%
67,799 particles
5.3%
Heterogeneous refinement
269,567 particles
Extract (bin1)
2D Classification
Heterogeneous refinement
681,520 particles
11,080 particles
1.6%
173,047 particles
25.4%
101,082 particles
14.8%
213,658 particles
31.4%
140,489 particles
20.6%
40,168 particles
5.9%
1,996 particles
0.3%
Sharpening
B
C
(Å)
2.5
3.5
4.5
2.51 Å
Cas8-HNH system full R-loop state
2.57 Å
Cas8-HNH system partial R-loop state
D
GSFSC Resolution 2.51Å
nomask (3.3 Å)
loose (2.8 Å)
tight (2.5 Å)
corrected (2.5 Å)
Cas8-HNH system full R-loop state
F
GSFSC Resolution 2.57Å
nomask (3.4 Å)
loose (2.9Å)
tight (2.6Å)
corrected (2.6Å)
Cas8-HNH system partial R-loop state
E
Elevation
Azimuth
# of images
Cas8-HNH system full R-loop state
G
Cas8-HNH system partial R-loop state
H
Cas7.1
Cas6
Cas7.2
Cas5
Cas7.3
Cas7.6
Cas7.5
Cas7.4
Cas8-HNH
target strand

Appendix Figure S6. Cryo-EM data processing workflow of Cas8-HNH system in partial and full R-loop states.

(A) Data processing workflow of Cas8-HNH system at partial and full R-loop states. **(B)** Local resolution analysis. The map of Cas8-HNH system at partial R-loop state colored by local resolution is shown. **(C)** Local resolution analysis. The map of Cas8-HNH system in full R-loop state colored by local resolution is shown. **(D)** Gold standard Fourier shell correlation carve. GSFSC carve of Cas8-HNH system at partial R-loop state was estimated using FSC=0.143 cutoff. **(E)** Orientation distributions of the reconstruction of Cas8-HNH system at partial R-loop. **(F)** Gold standard Fourier shell correlation carve. GSFSC carve of Cas8-HNH system at full R-loop was estimated using FSC=0.143 cutoff. **(G)** Orientation distributions of the reconstruction of Cas8-HNH system at full R-loop. **(H)** The positively charged TS binding groove formed by interlocked Cas7 subunits.

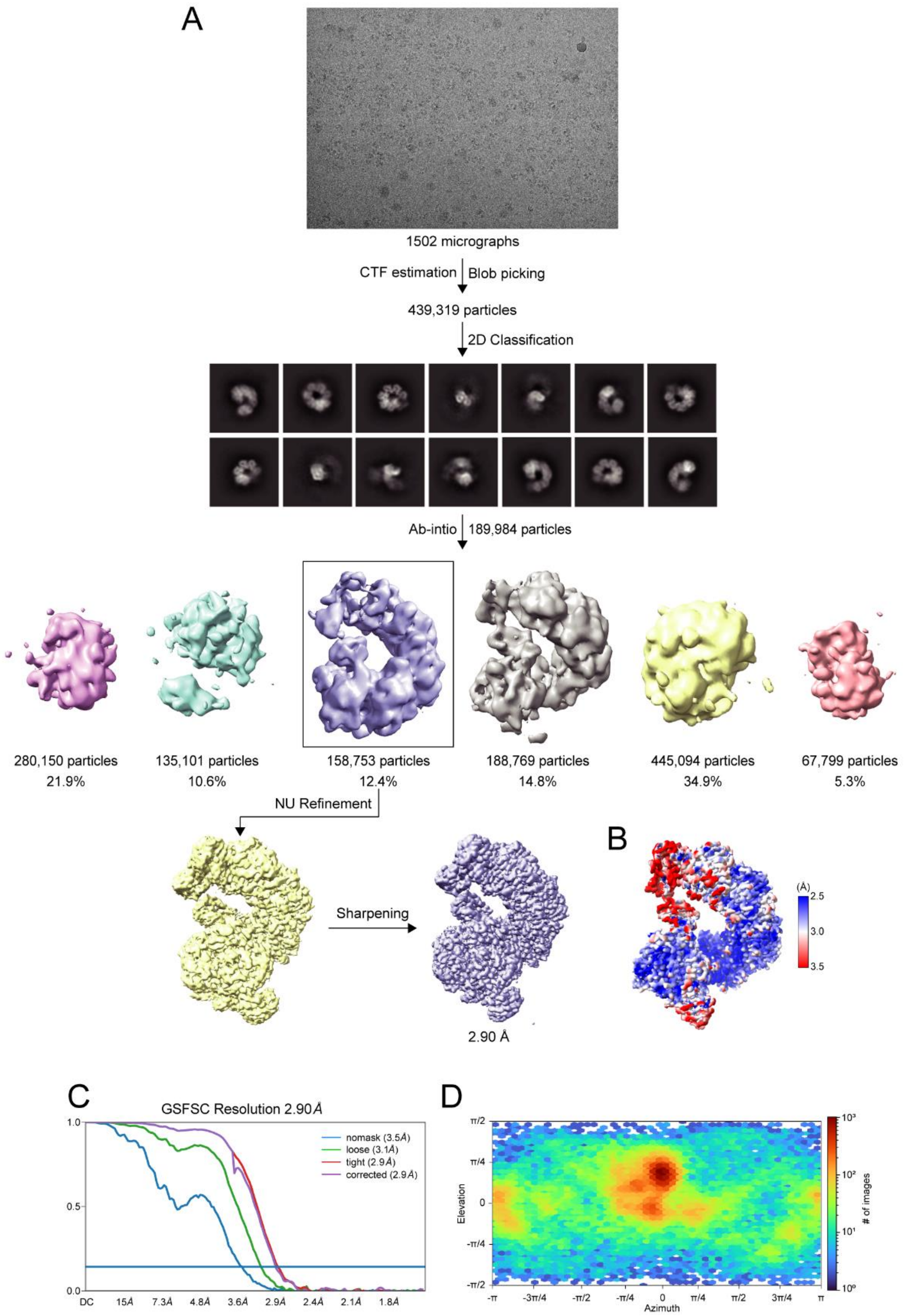

Appendix Figure S7. Cryo-EM data processing workflow of type I-F Cas8-HNH system in ssDNA-bound state.

(A) Data processing workflow of type I-F Cas8-HNH system in ssDNA-bound state. **(B)** Local resolution analysis. The map of Cas8-HNH system at ssDNA-bound state colored by local resolution is shown. **(C)** Gold standard Fourier shell correlation carve. GSFSC carve of Cas8-HNH system at ssDNA-bound state was estimated using FSC=0.143 cutoff. **(D)** Orientation distributions of the reconstruction of Cas8-HNH system at ssDNA-bound state.

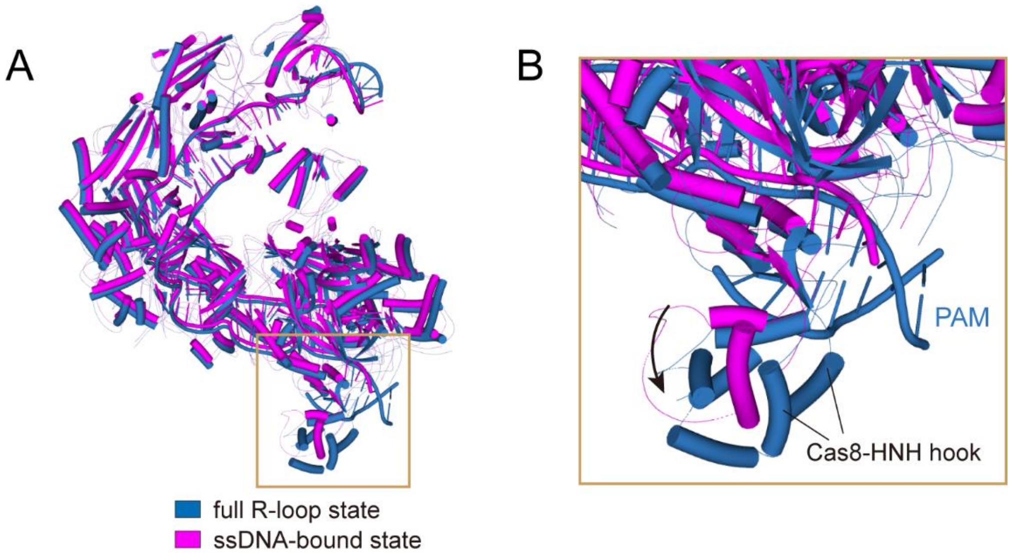

Appendix Figure S8. Structural differences between the full R-loop and ssDNA-bound states. (A) Superposition of the type I-F Cas8-HNH system in the full R-loop (sky blue) and ssDNA-bound states (magenta). **(B)** Close-up view of the conformational changes in the hook region.

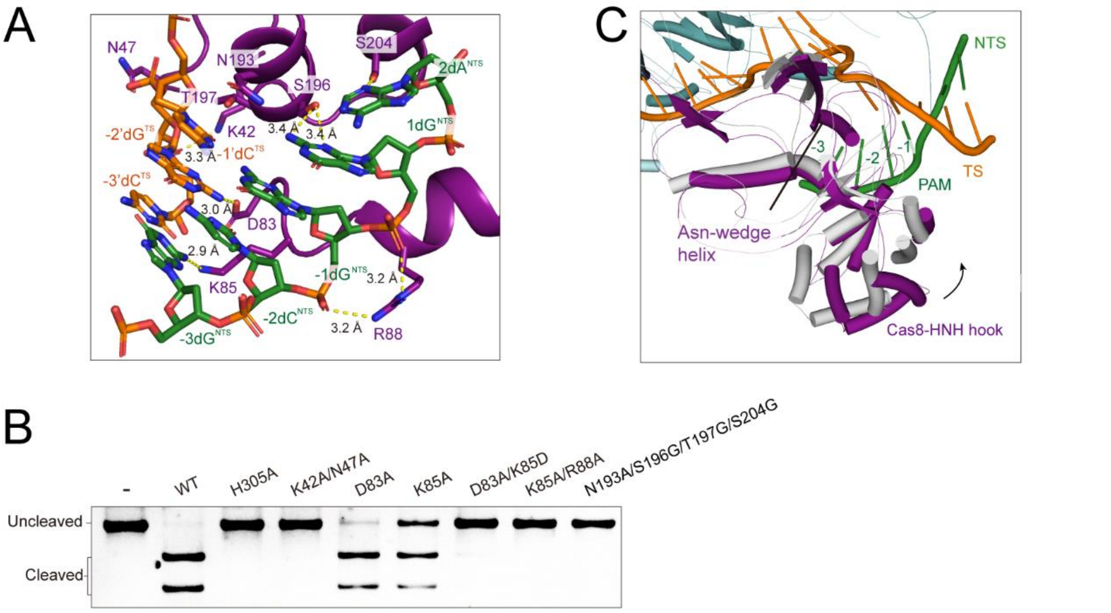

Appendix Figure S9. PAM recognition mechanism of type I-F Cas8-HNH system.

(A) PAM recognition mode of type I-F Cas8-HNH system. Key residues responsible for PAM interaction on the Cas8-HNH protein are shown as sticks. **(B)** In vitro DNA cleavage assay of the WT and mutant Cas8-HNH systems. Key PAM-interacting residues from Cas8-HNH protein were mutated. The gel is representative of three individual experiments. **(C)** Conformational changes of Cas8-HNH protein upon target DNA loaded. The Cas8-HNH hook region in RNP complex is shown in gray. In contrast to the Cascade-crRNA complex, the Cas8 hook (purple) rotates inward at the target-bound state. The black arrow indicates the movement of the Cas8 hook.

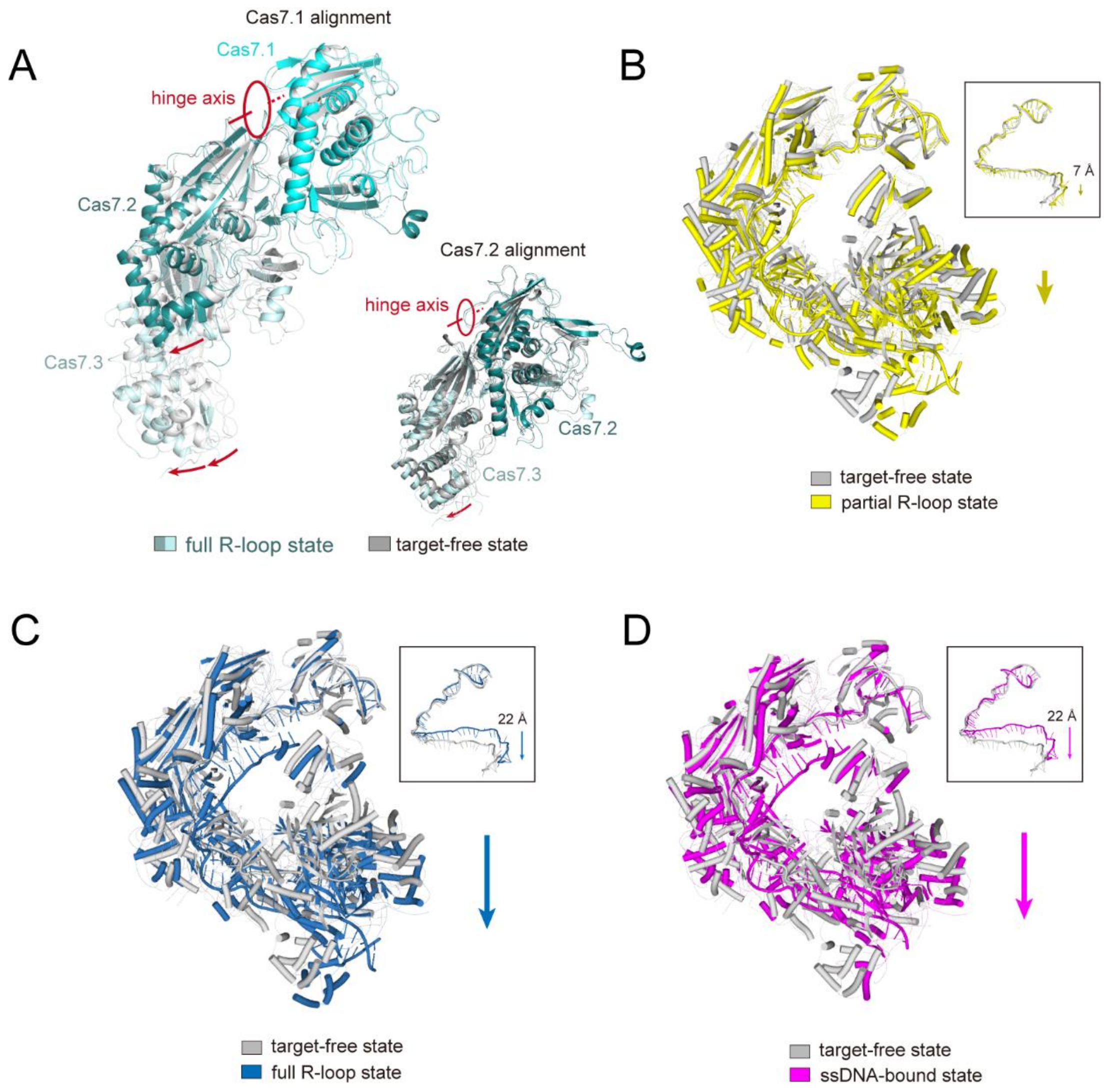

Appendix Figure S10. Full R-loop formation is essential for the activation of type I-F Cas8-HNH system.

(A) Structural comparison of Cas7.1-3 and Cas7.2-3 (inset panel) subunits in full R-loop (cyan and deep teal) and target-free (gray) states. Rotations are indicated as red arrows. **(B-D)** Structure alignments of type I-F Cas8-HNH system at target-free (gray) state with partial R-loop (yellow), full R-loop (sky blue), and ssDNA-bound (magenta) states, respectively. Conformational changes of the Cascade complex upon partial R-loop formation, full R-loop formation, and ssDNA binding are indicated by yellow, sky blue, and magenta arrows, respectively.

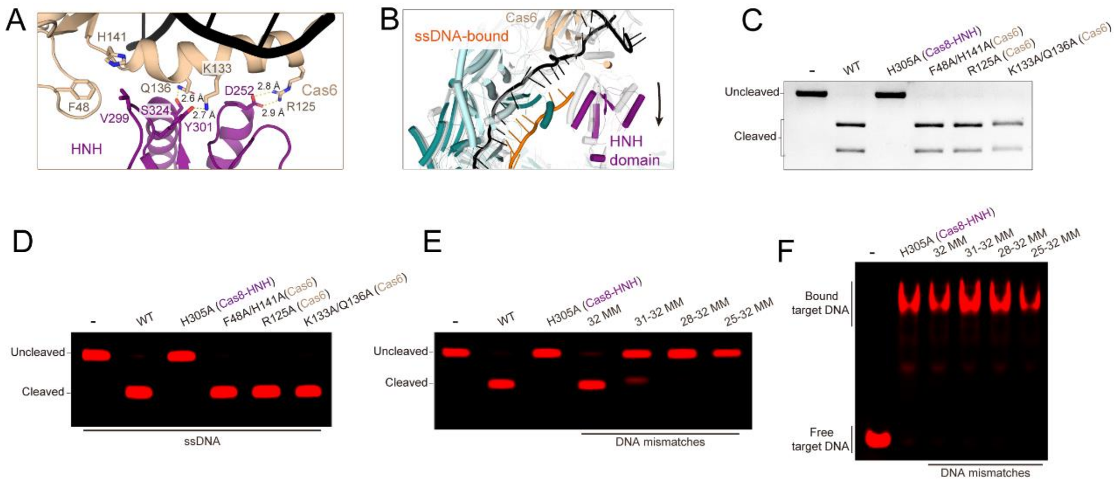

Appendix Figure S11. Complete base pairing is vital to the activation of HNH nuclease.

(A) Detailed insights into the contacts between the HNH domain and Cas6 subunit in the RNP complex. Key interacting residues are shown as sticks. **(B)** Structure alignments of target-free (gray) and ssDNA-bound states. HNH domain displaces from Cas6 subunit upon ssDNA binding. **(C)** In vitro dsDNA cleavage assay of WT and mutant Cas8-HNH Cascade. Key residues responsible for interacting with HNH domain on Cas6 subunit were mutated. **(D)** In vitro ssDNA cleavage assay of WT and mutant Cas8-HNH Cascade utilizing 3'-Cy5 labeled ssDNA. **(E)** Substrate ssDNA cleavage assay with various mismatched DNA utilizing 3'-Cy5 labeled ssDNA. **(F)** DNA EMSA of various mismatched dsDNA with 3'-Cy5 fluorophore. The gels are representative of three independent experiments.

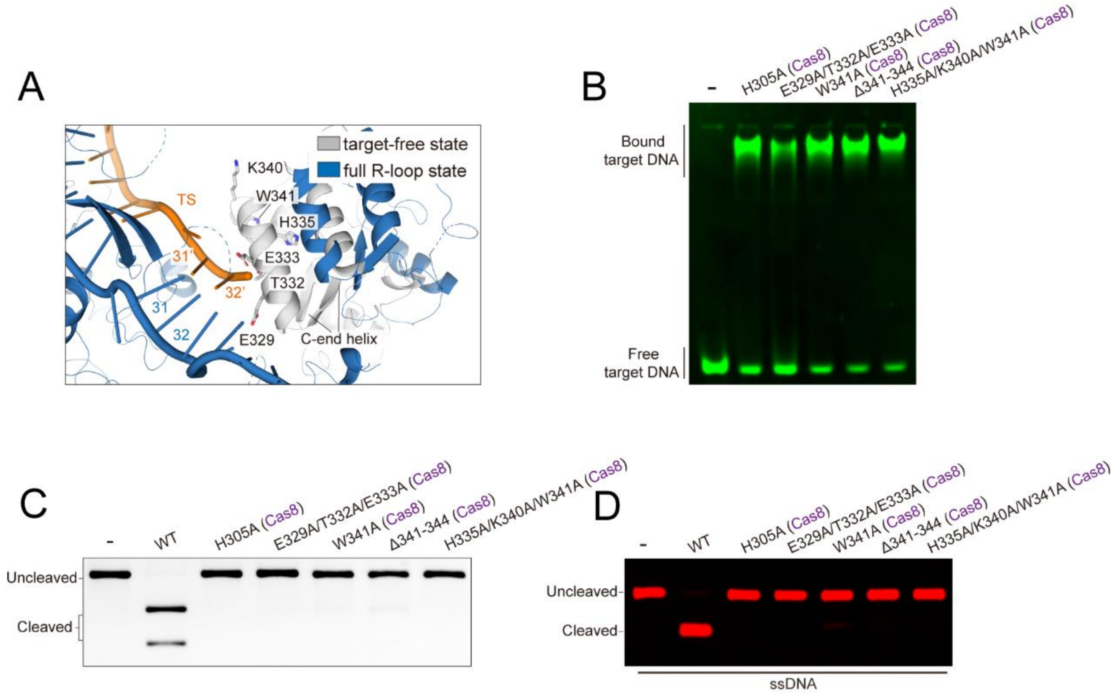

Appendix Figure S12. The C-end helix of HNH domain plays an important role in target recognition and cleavage.

(A) Close-up view of the C-terminal helix in the target free and full R-loop states. The key residues from the C-terminal helix are shown as sticks. **(B)** DNA EMSA of WT and mutated Cas8-HNH Cascade with 5'-Cy3 labeled dsDNA, respectively. All the gels are representative of three independent experiments. **(C)** In vitro dsDNA cleavage assay of WT and mutated Cas8-HNH Cascade. The putative residues involved in target DNA recognition were mutated. **(D)** The ssDNA cleavage assay of WT and mutated Cas8-HNH Cascade with 3'-Cy5 labeled ssDNA.

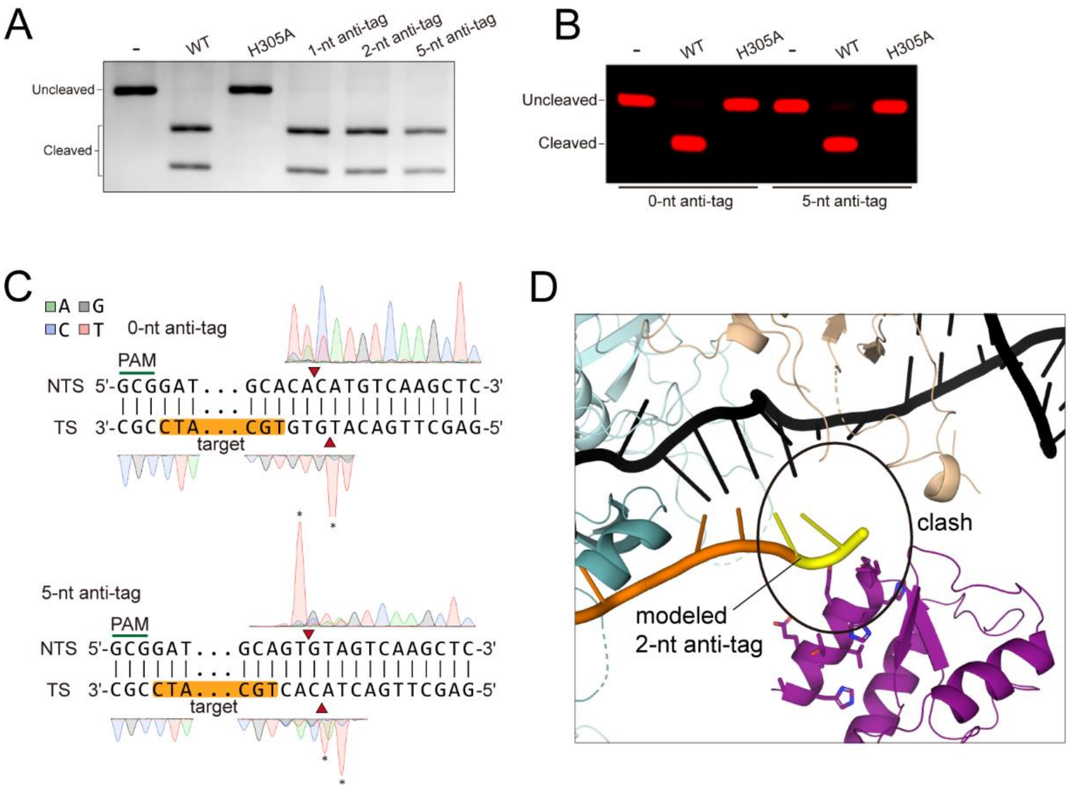

Appendix Figure S13. DNA cleavage of type I-F Cas8-HNH system with different anti-tag DNA.

(A) In vitro DNA cleavage assay of Cas8-HNH system with different anti-tag DNA. **(B)** In vitro ssDNA cleavage assay utilizing different anti-tag sequences. **(C)** Analysis of the cleavage products with 0-nt and 5-nt anti-tags of Cas8-HNH system utilizing Sanger sequencing, respectively. The red triangle indicates the cleavage site. **(D)** The HNH domain in full R-loop state clashes with the modeled 2-nt anti-tag, indicating that the anti-tag sequence cannot form complementary base pairs with the crRNA after the spacer region. The modeled nucleotides are colored yellow.

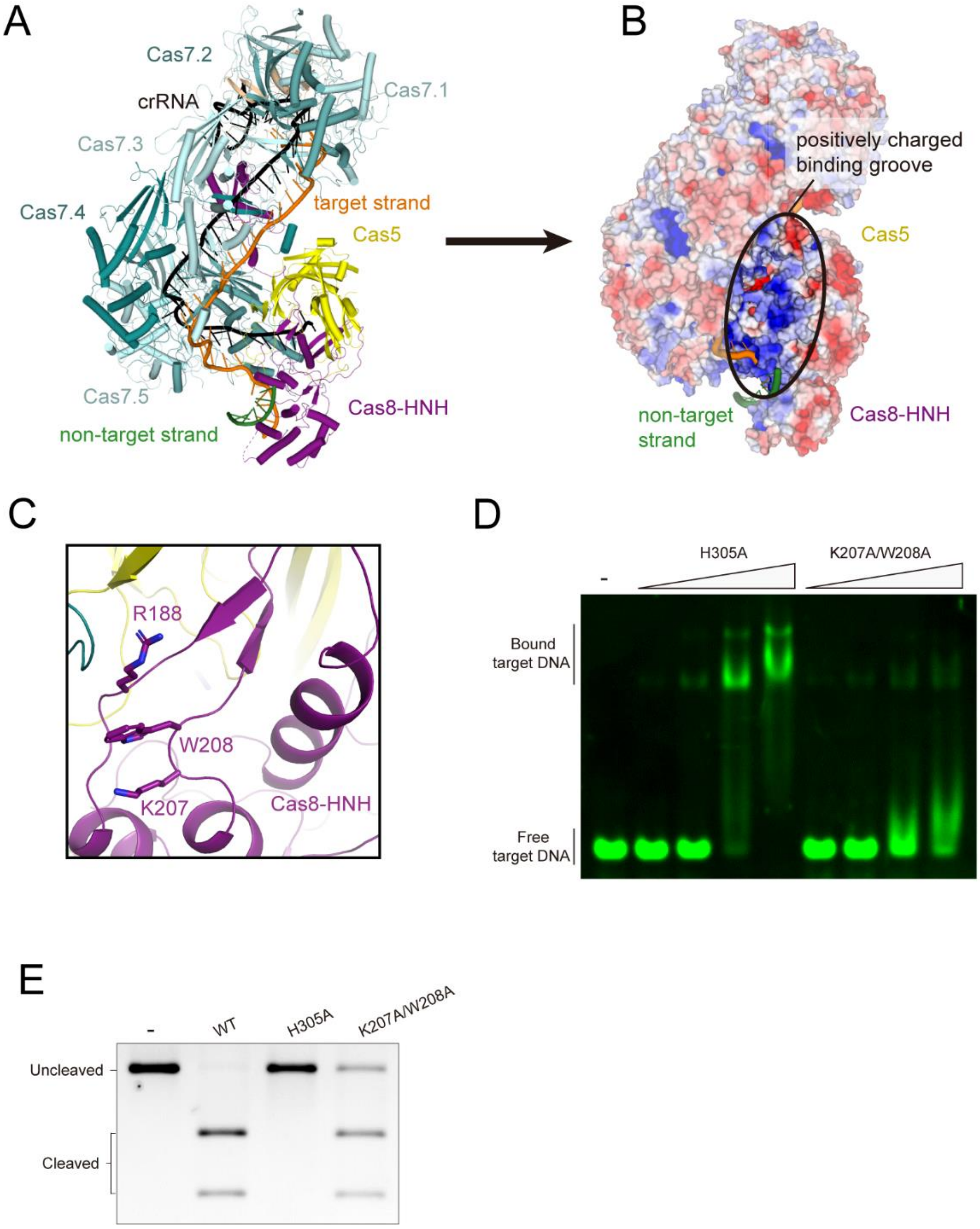

Appendix Figure S14. A positively charged binding groove is responsible for NTS binding. **(A)** Atomic model of target-bound Cas8-HNH Cascade. The NTS is colored green. **(B)** Electrostatic surface potential of Cas8-HNH Cascade complex. The positively charged binding pocket on the Cas8-HNH complex marked by a black circle is responsible for NTS binding. **(C)** Close-up views of the putative NTS binding groove on the Cas8-HNH complex. Key residues from the Cas8-HNH subunit forming the positively charged binding groove are shown as sticks. **(D)** DNA Electrophoresis mobility shift assay (EMSA) with increasing concentration of mutated Cas8-HNH Cascade complex. The 5'-Cy3 labeled dsDNA was used as a probe. The gel is representative of three independent experiments. **(E)** In vitro DNA cleavage assay of WT and mutated Cas8-HNH complex. The result represents three repeat experiments.

Appendix Table S1. Cryo-EM data collection, refinement and validation statistics

	Cas8-HNH Cascade-crRNA complex	Cas8-HNH Cascade-crRNA-dsDNA complex		Cas8-HNH Cascade-crRNA-ssDNA complex
	Target-free state (EMDB-60017) (PDB 8ZDY)	Full R-loop state (EMDB-39706) (PDB 8Z0K)	Partial R-loop state (EMDB-39707) (PDB 8Z0L)	ssDNA-bound state (EMDB-60279) (PDB 8ZNR)
**Data collection and processing**				
Magnification	165,000	165,000	165,000	165,000
Voltage (kV)	300	300	300	300
Electron exposure (e–/Å^2)	60	60	60	50
Defocus range (μm)	-1.2 to -1.6	-1.2 to -1.6	-1.2 to -1.6	-1.2 to -1.8
Pixel size (Å)	0.856	0.750	0.750	0.808
Symmetry imposed	*C1*	*C1*	*C1*	*C1*
Initial particle images (no.)	750,550	1,645,462	1,645,462	525,787
Final particle images (no.)	220,116	173,047	140,489	77,904
Map resolution (Å)	3.61	2.51	2.57	2.90
FSC threshold	0.143	0.143	0.143	0.143
Map resolution range (Å)	3.2-3.8	2.5-4.5	2.5-4.5	2.5-3.5
**Refinement**				
Initial model used (PDB code)	AlphaFold	AlphaFold	AlphaFold	AlphaFold
Model resolution (Å)	2.0	2.1	2.1	2.0
FSC threshold	0.5	0.5	0.5	0.5
Model resolution range (Å)	1.7-3.7	1.6-2.8	1.6-2.9	1.6-3.2
Map sharpening *B* factor (Å^2)	-147.8	-100.5	-100.1	-83.1
Model composition				
Non-hydrogen atoms	21553	23048	22930	22605
Protein residues	2629	2646	2643	2607
Nucleotides	58	102	97	91
B factors (Å^2)				
Protein	76.91	61.15	66.26	67.97
Nucleotide	83.26	77.39	86.93	70.15
R.m.s. deviations				
Bond lengths (Å)	0.010	0.007	0.008	0.007
Bond angles (°)	1.518	1.262	1.303	1.170
Validation				
MolProbity score	2.21	1.81	1.87	1.92
Clashscore	10.40	5.45	6.57	6.59
Poor rotamers (%)	1.97	1.43	1.26	1.40
Ramachandran plot				
Favored (%)	92.95	94.01	93.39	92.98
Allowed (%)	6.89	5.26	6.23	6.59
Disallowed (%)	0.15	0.73	0.38	0.43
