# Supplementary figures and images for: Structural basis for the type I-F Cas8-HNH system
Source: EMBO J. 2024 Sep 9;43(20):4656–67. doi: 10.1038/s44318-024-00229-8 (PMC11480323; doi:10.1038/s44318-024-00229-8)

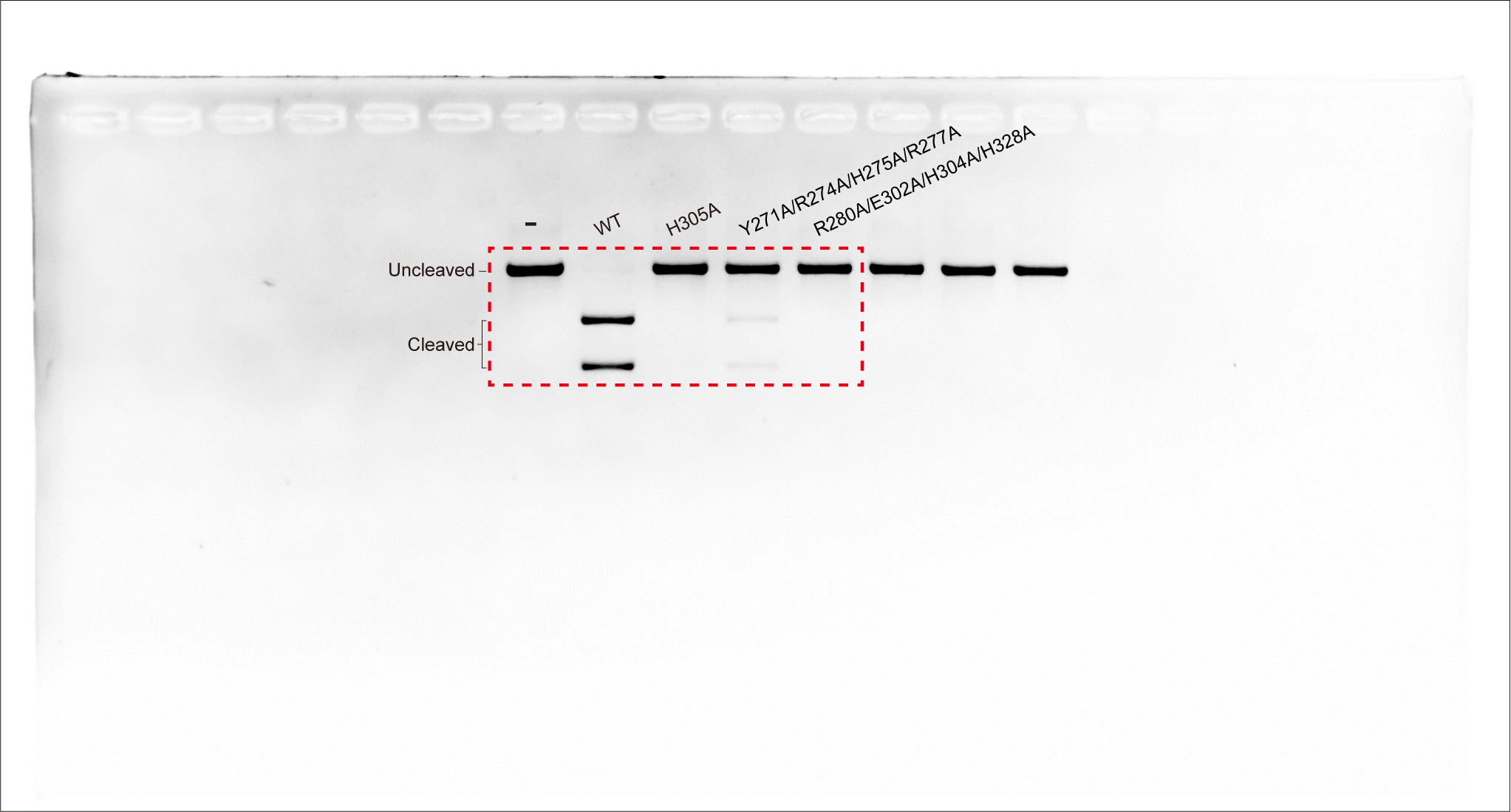

Supplement: Supplementary file 3 — Source data Fig. 1 [file 44318_2024_229_MOESM3_ESM.zip › Figure 1/1G/1G.tif]

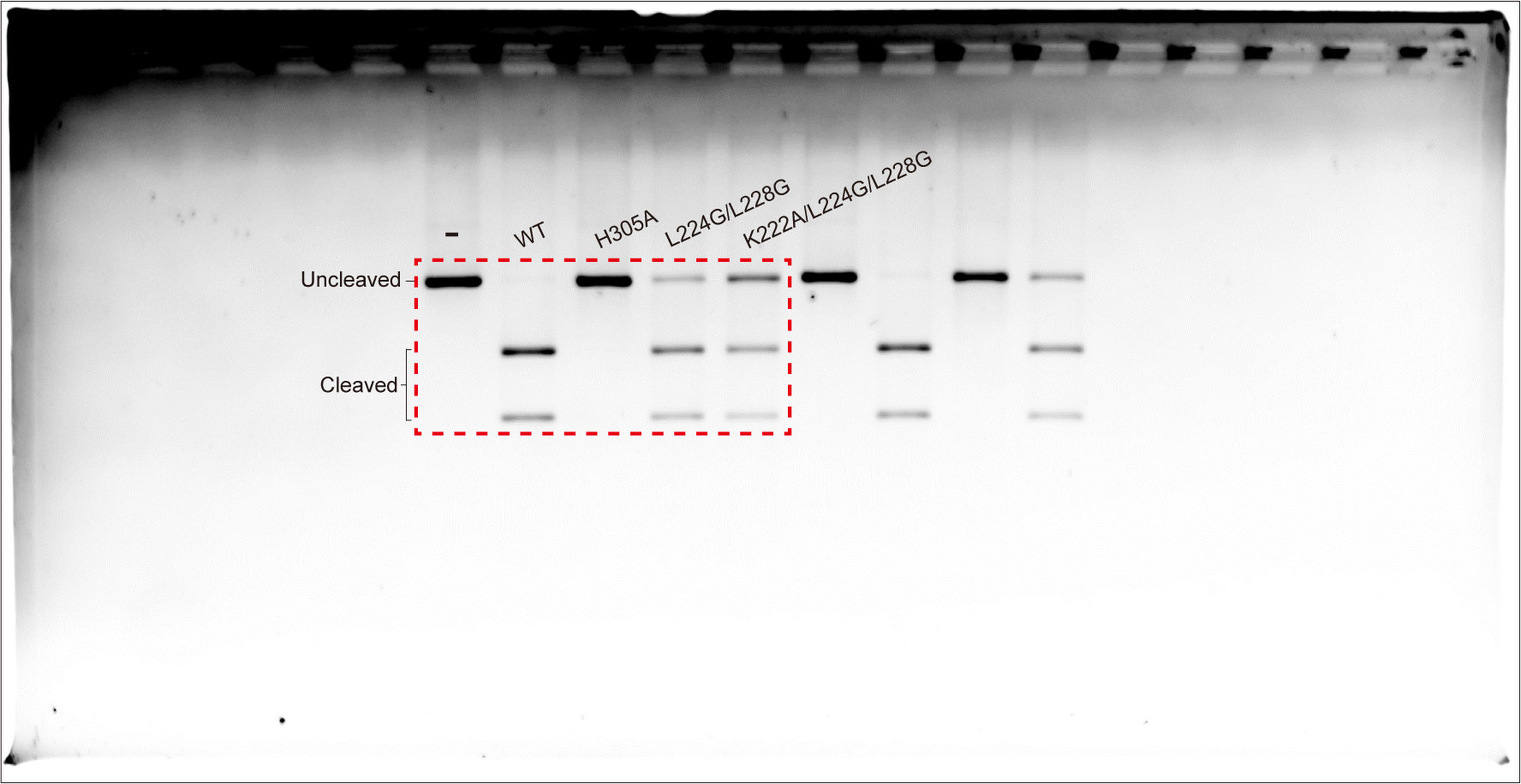

Supplement: Supplementary file 4 — Source data Fig. 3 [file 44318_2024_229_MOESM4_ESM.zip › Figure 3/3C/3C.tif]

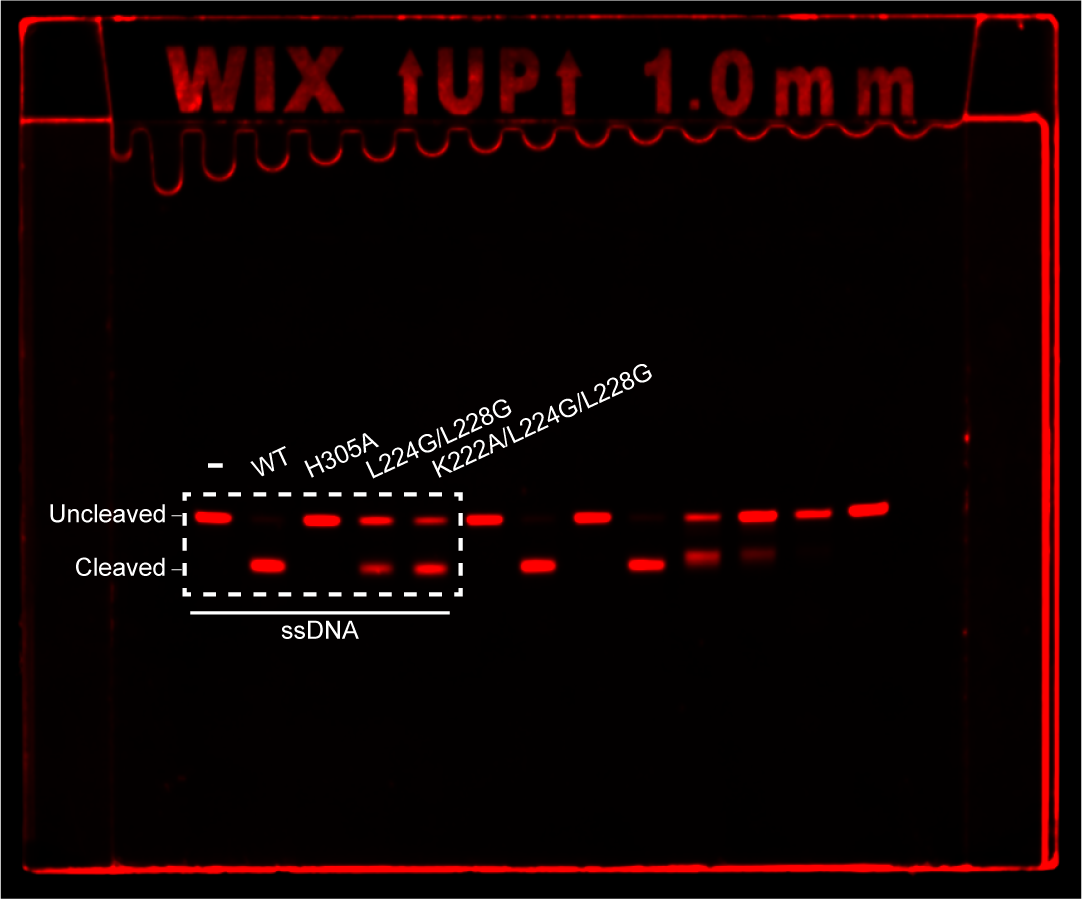

Supplement: Supplementary file 4 — Source data Fig. 3 [file 44318_2024_229_MOESM4_ESM.zip › Figure 3/3D/3D.tif]

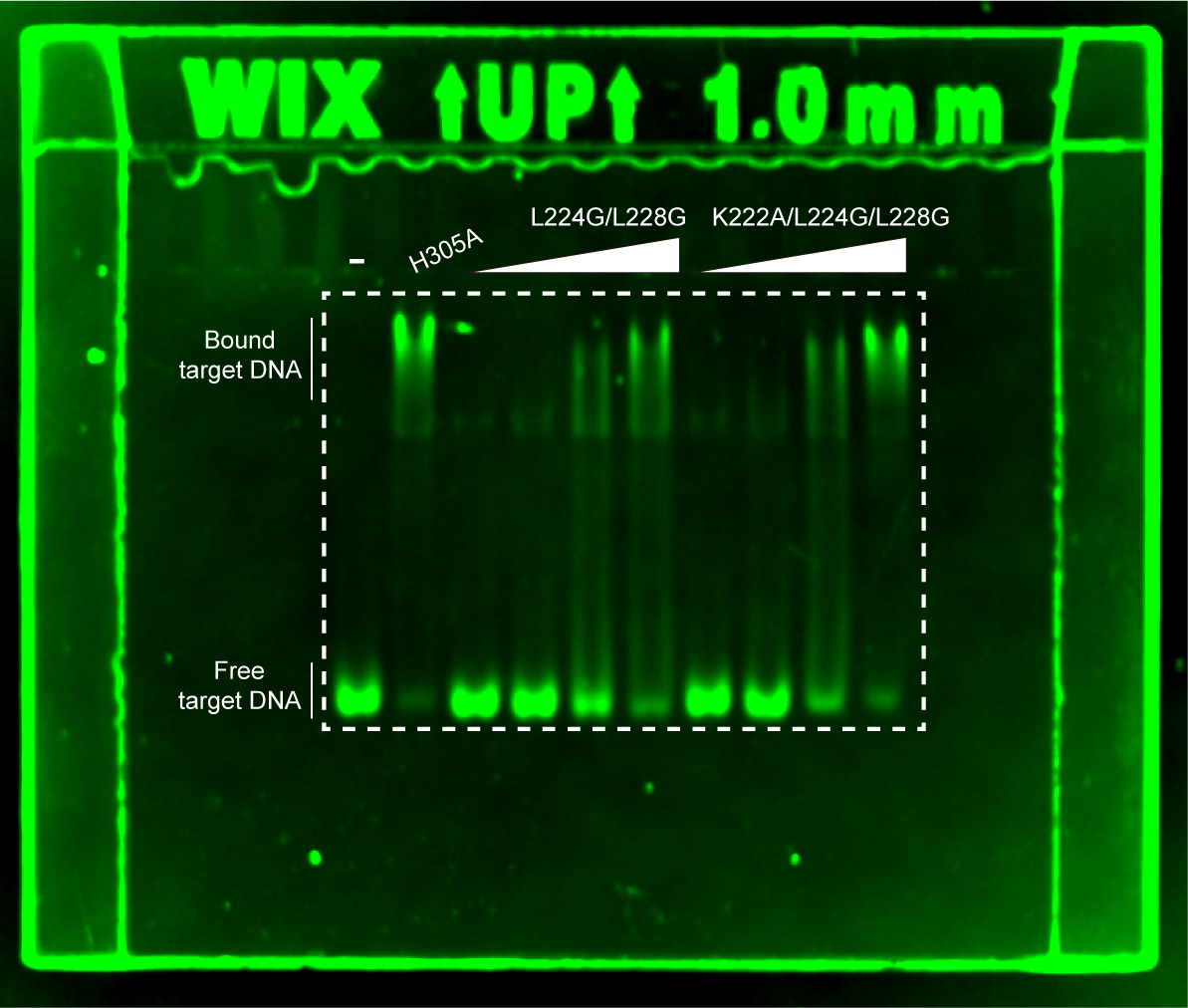

Supplement: Supplementary file 4 — Source data Fig. 3 [file 44318_2024_229_MOESM4_ESM.zip › Figure 3/3E/3E.tif]

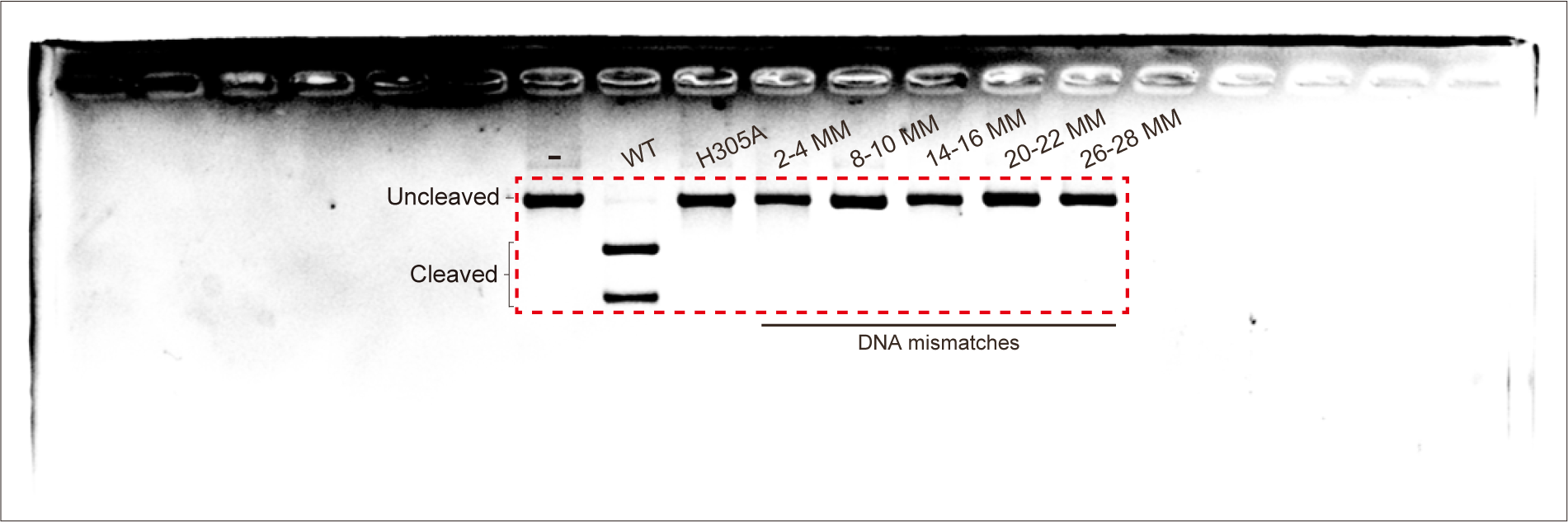

Supplement: Supplementary file 5 — Source data Fig. 4 [file 44318_2024_229_MOESM5_ESM.zip › Figure 4/4C/4C.tif]

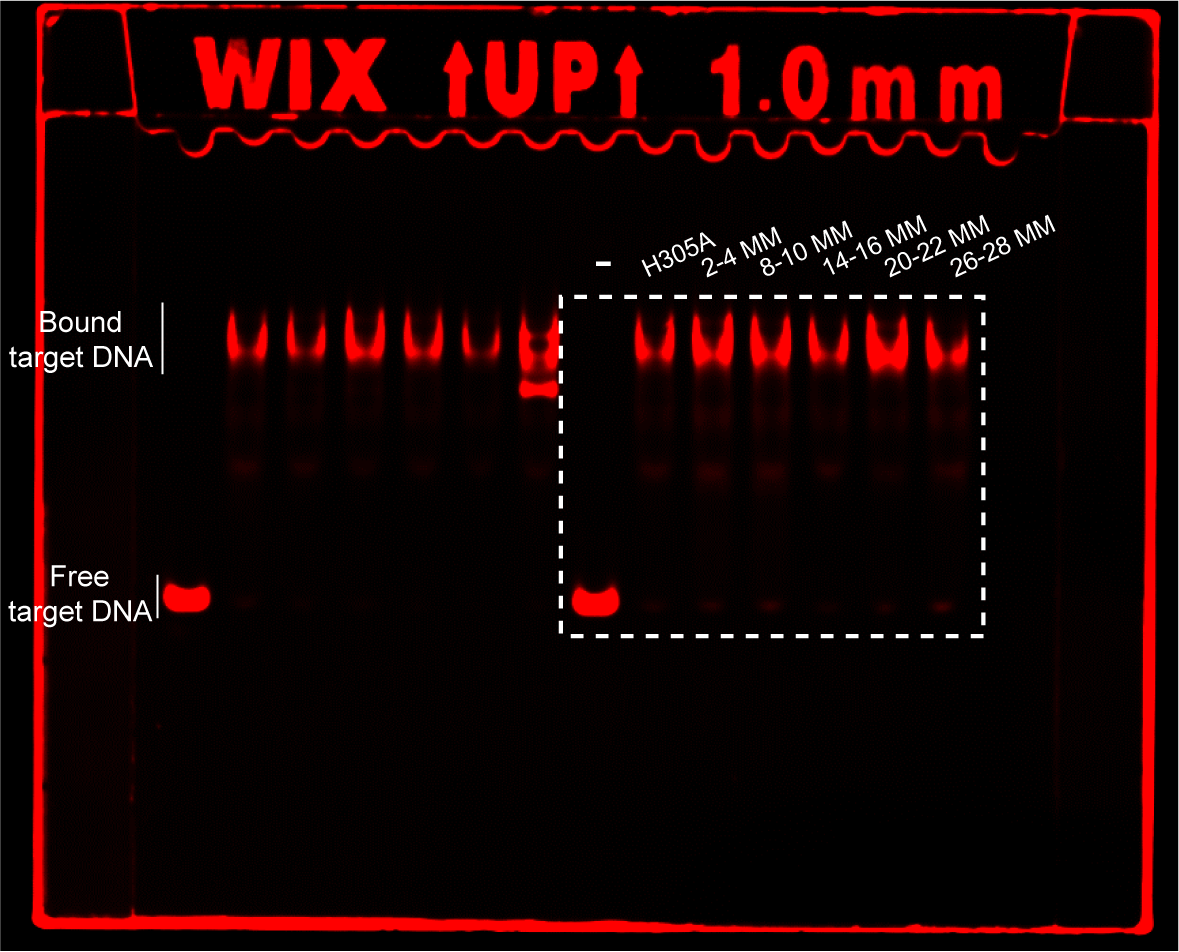

Supplement: Supplementary file 5 — Source data Fig. 4 [file 44318_2024_229_MOESM5_ESM.zip › Figure 4/4D/4D.tif]

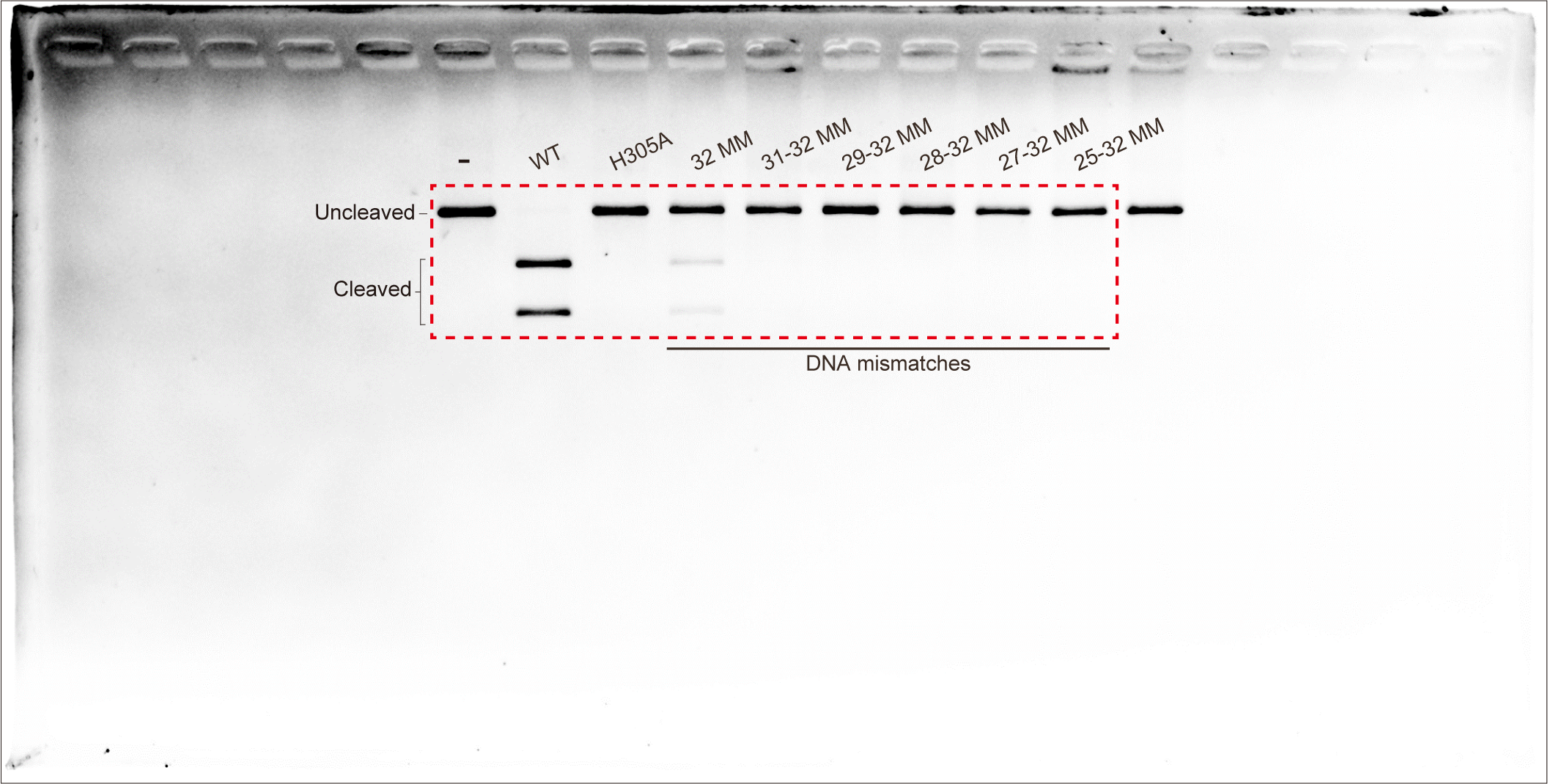

Supplement: Supplementary file 6 — Source data Fig. 5 [file 44318_2024_229_MOESM6_ESM.zip › Figure 5/5D/5D.tif]
